# Characterization of Nerolidol Synthase (VsNES1) from *Veronicastrum sibiricum* via Transcriptome Analysis

**DOI:** 10.3390/plants14243813

**Published:** 2025-12-15

**Authors:** Zhi-Ying Wang, Xiang-Xiang Ren, Yan-Bo Huang, Xue Li, Hong-Peng Chen

**Affiliations:** 1 School of Traditional Chinese Medicine, Guangdong Pharmaceutical University, Guangzhou 510006, China; 2 Shanghai Key Laboratory of Plant Functional Genomics and Resources, Shanghai Chenshan Bo-Tanical Garden, Chenshan Science Research Center, CAS Center for Excellence in Molecular Plant Sciences (CEMPS), Chinese Academy of Sciences (CAS), Shanghai 201602, China

**Keywords:** *V. sibiricum*, terpene synthase, (E)-nerolidol synthase, synthetic biology

## Abstract

*Veronicastrum sibiricum* (L.) Pennell, a species within the Plantaginaceae family, has a history of traditional application in addressing conditions such as abdominal pain, common cold, sore throat, parotitis, rheumatic discomfort, and snakebite. The plant produces diverse bioactive constituents, including phenylpropanoids, essential oils, flavonoids, and terpenoids. Terpenoids, generated via terpene synthases (TPSs), are of particular interest due to their pharmacological properties. Nevertheless, TPS enzymes in *V. sibiricum* have not been thoroughly investigated. In this research, a transcriptomic strategy was employed to isolate and profile TPS genes from *V. sibiricum*. Sequencing of the transcriptome produced 107,929 unigenes, among which 42,976 were functionally annotated using public databases. KEGG pathway examination revealed 264 genes associated with terpenoid metabolism, including 12 putative *VsTPS* genes harboring characteristic TPS domains. From these, *VsTPS1* was successfully cloned. Functional characterization established that VsTPS1 operates as a bifunctional enzyme: in vitro, it catalyzes the conversion of FPP to (E)-nerolidol and, to a lesser extent, GPP to linalool. When expressed transiently in *Nicotiana benthamiana*, however, only (E)-nerolidol was detected, supporting its cytosolic localization and substrate specificity toward FPP. Accordingly, this sesquiterpene synthase was redesignated *VsNES1*. Co-expression of *VsNES1* with *HMGR* in *N. benthamiana* markedly increased (E)-nerolidol yields, illustrating an effective strategy for heterologous production. These findings deepen our understanding of the TPS family in medicinal plant *V. sibiricum* and enable future biotechnological exploitation of terpenoid production in heterogenous plant cells.

## 1. Introduction

*V. sibiricum* is a plant of significant standing in traditional Chinese medicine, renowned for its multifaceted pharmacological activities, including anti-inflammatory, analgesic, antitumor, and anti-cancer effects [1,2,3]. The historical application of Veronicastrum species in medicine has undergone notable taxonomic shifts. Archival records, particularly from the Song Dynasty, emphasize the use of Plantaginaceae varieties for conditions such as edema and for detoxification purposes. Contemporary pharmacological investigations have since validated these traditional uses, demonstrating that iridoid glycosides [4], flavonoid glycosides [5], and diterpenes derived from herbaceous Veroni-castrum exhibit superior anti-inflammatory and analgesic properties compared to alternatives from other families [1,6].

The therapeutic potential of this herb is largely attributed to its terpenoid constituents. However, the clinical translation and industrial scale-up of these compounds are severely hampered by their low abundance in the native plant and the economically challenging extraction processes [7]. Synthetic biology has emerged as a transformative approach to overcome such supply limitations, leveraging engineered microbial systems as efficient “cell factories” for the production of complex natural products [8,9,10]. Nevertheless, the successful application of this strategy is contingent upon a comprehensive elucidation of the biosynthetic pathways, which for *V. sibiricum* remains a fundamental challenge. The lack of foundational genomic and transcriptomic data has left the terpenoid biosynthesis machinery in this species largely unexplored, thereby impeding any meaningful synthetic biology efforts [11]. This gap is particularly pronounced for the biosynthesis of sesquiterpenes like (E)-nerolidol, where pathway discovery in non-model plants is often the rate-limiting step [12,13,14].

In plants, the biosynthesis of terpenoids proceeds via two distinct pathways—the mevalonate (MVA) and methylerythritol-4-phosphate (MEP) pathways—with the structural diversity of the final products being sculpted by terpene synthases (TPSs) and a suite of downstream modifying enzymes, such as cytochrome P450s and glycosyltransferases [15,16,17]. While this general framework is well-established, the specific genetic components and enzymatic players within *V. sibiricum* are entirely unknown. This critical knowledge gap has stalled efforts to map its biosynthetic potential and reconstruct the pathways in heterologous hosts [18,19,20].

To bridge this gap, we report the first comprehensive transcriptome analysis of *V. sibiricum*. This genomic resource served as a foundation for the systematic identification of genes putatively involved in terpenoid biosynthesis. Our study specifically focused on the terpene synthase (TPS) family, for which we conducted phylogenetic and tissue-specific expression profiling. A prime candidate, VsTPS1, was subsequently characterized through a combination of in vitro enzymatic assays and in planta tobacco transient expression. Our functional studies unequivocally identified VsTPS1 as a nerolidol synthase, leading to its reclassification as *VsNES1*. These findings not only pinpoint a key catalyst in the sesquiterpene biosynthesis pathway of *V. sibiricum* but also establish a foundational platform for the sustainable bioproduction of (E)-nerolidol.

## 2. Results

### 2.1. Transcriptome Assembly and Reproducibility Analysis Reveals Tissue-Specific Expression Patterns

**Figure 1:**
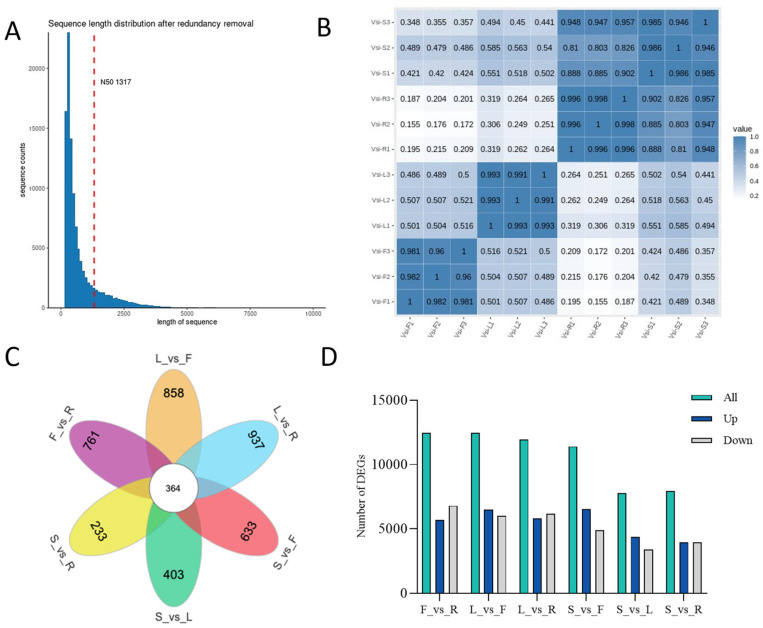
Analysis of transcriptome sequencing data (F, flower; L, leaf; R, root; S, stem). (**A**) Distribution of Unigene lengths. The horizontal axis represents the length of Unigenes, and the vertical axis indicates the number of Unigenes within the corresponding length range. The red dashed line indicates the N50 length threshold. (**B**) Density distribution of FPKM values in various samples. The x-axis represents the log10-transformed FPKM values, and the y-axis represents the gene density. Different colors indicate different samples. (**C**) Pairwise comparisons among root, stem, leaf, and inflorescence showing the number of differentially expressed genes (DEGs) and the number of intersecting genes. (**D**) Pairwise comparisons among F, L, R, and S. The x-axis indicates the comparison groups, and the y-axis indicates the number of DEGs. Green represents the total number of DEGs between two tissues, blue represents up-regulated genes, and gray represents down-regulated genes.

Initial sequencing statistics are provided in Supplementary Table S6. Read counts across root, stem, leaf, and inflorescence samples showed generally consistent yields. Raw reads were quality-filtered and adapter-trimmed using fastp to eliminate sequences that could affect subsequent analyses. After processing, all samples produced clean reads with Q20 and Q30 scores above 98.8% and 96.7%, respectively, confirming high base-call accuracy and overall sequencing quality suitable for downstream processing. Transcript assembly was carried out with Trinity (v2.11.0), generating 274,076 transcripts with an N50 value of 1676 bp. Redundancy was reduced using cd-hit, where the longest transcript from each cluster was designated as a Unigene, yielding a final set of 107,929 Unigenes. These unigenes varied in length from 201 bp to 15,423 bp, with a mean length of 794.6 bp and an N50 of 1317 bp. The distribution of unigene lengths is presented in Figure 1A.

Reproducibility among the 12 samples (representing root, stem, leaf, and inflorescence tissues) was evaluated using Pearson correlation. Biological replicates within the same tissue type showed correlations above 0.9 (Figure 1B), indicating high experimental consistency. Additionally, stem and root samples exhibited notably high correlation, implying shared expression profiles between these two tissue types. As shown in the Venn diagram (Figure 1C), the number of unique differentially expressed genes (DEGs) between root and stem was the smallest (only 233), suggesting relatively similar functional profiles between these two tissues, which is consistent with the sample correlation results shown in Figure 1B. Additionally, 364 DEGs were common across all compared tissues. Further comparative analysis (Figure 1D) revealed that the greatest number of DEGs was observed between inflorescence and root (12,465 DEGs; 5677 up- and 6788 down-regulated in root) and between inflorescence and leaf (12,472 DEGs; 6482 up- and 5990 down-regulated in leaf). In contrast, the smallest differences were detected between stem and root (7937 DEGs; 3973 up- and 3964 down-regulated in root) and between stem and leaf (7769 DEGs; 4367 up- and 3402 down-regulated in leaf).

### 2.2. KEGG Pathway Enrichment Analysis of Differentially Expressed Unigenes

Among the differentially expressed genes across various tissues, a total of four pathways related to terpenoid biosynthesis were annotated. The distribution of gene counts in these pathways is shown in Supplementary Figure S1A. Based on the functional annotation of unigenes from the transcriptome, 83 genes were identified to be involved in the upstream biosynthesis of terpenoids. Among these, 21 genes participate in the MEP pathway, including 6 DXS, 3 DXR, 1 MCT, 3 CMK, and others. Mean-while, 37 genes are involved in the MVA pathway, including 11 AACT, 12 HMGS, 5 HMGR, 2 MK, among others. In addition, 3 IDI genes were identified, which coding protein catalyze the interconversion of IPP and DMAPP. As key enzymes for terpenoid skeleton formation, 3 FPPS, 5 GPPS, and 14 GGPPS genes were also identified. Furthermore, 31 genes were associated with monoterpene synthesis, 32 with sesquiterpene and triterpene synthesis, 24 with diterpene synthesis, and 58 with carotenoid biosynthesis (Supplementary Table S7).

**Figure 2:**
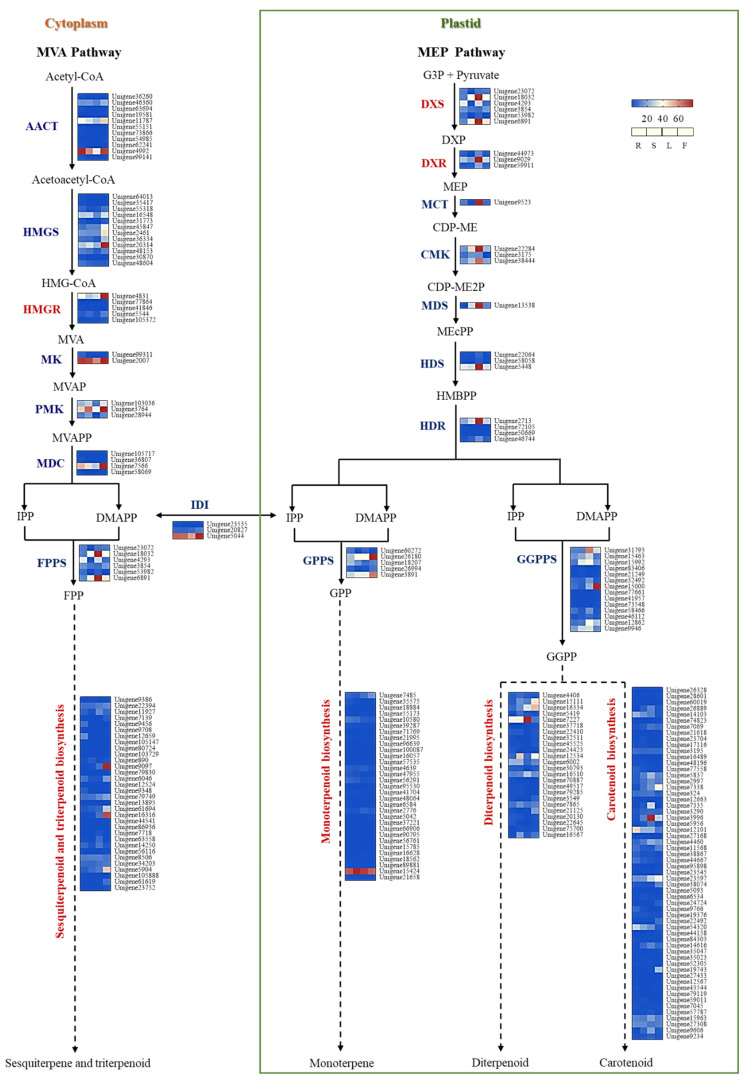
Expression profiles of terpene biosynthesis-related genes. Expression patterns of genes enriched in the MVA and MEP pathways. The MEP and MVA pathways are shown inside and outside of the green box, respectively. Expression levels in root, stem, leaf, and inflorescence (from left to right) are shown, with red indicating higher expression and blue indicating lower expression (F, flower; L, leaf; R, root; S, stem).

Notably, the number of genes involved in the MVA pathway was 16 greater than those in the MEP pathway. Expression profiling revealed that several genes in the MEP pathway were highly expressed in leaves, possibly supporting chlorophyll metabolism, whereas multiple genes in the MVA pathway showed high expression in inflorescences (Figure 2). Using an HMM model, conserved TPS domains were screened among genes annotated in terpenoid biosynthesis pathways, leading to the identification of 12 candidate VsTPS genes, among which only VsTPS1 was successfully cloned. Differential expression analysis of these VsTPS genes indicated that most were highly expressed in inflorescences, suggesting a potential role in floral scent formation. Three genes showed high expression in leaves, three in roots, and only one was highly expressed in stems—consistent with our earlier finding that the stem contains the lowest abundance of terpenoids among all tissues (Supplementary Figure S1B).

### 2.3. Molecular Characterization and Classification of VsTPS1

Physicochemical analysis revealed that VsTPS1 is a 532-amino-acid protein with a molecular weight of 61.04 kDa. It is an acidic, hydrophilic, and stable protein. Subcellular localization predictions indicated the absence of both transmembrane domains and a signal peptide, which is consistent with a cytoplasmic localization. Secondary structure was dominated by alpha-helices (68.61%) and random coils (30.08%) (Supplementary Table S8 and Supplementary Figure S3A–C). Multiple sequence alignment with TPS homologs from *Antirrhinum majus*, *Osmanthus fragrans*, *Olea europaea* var. *sylvestris*, *Actinidia polygama*, and *Camellia sinensis* is provided in Supplementary Figure S2.

**Figure 3:**
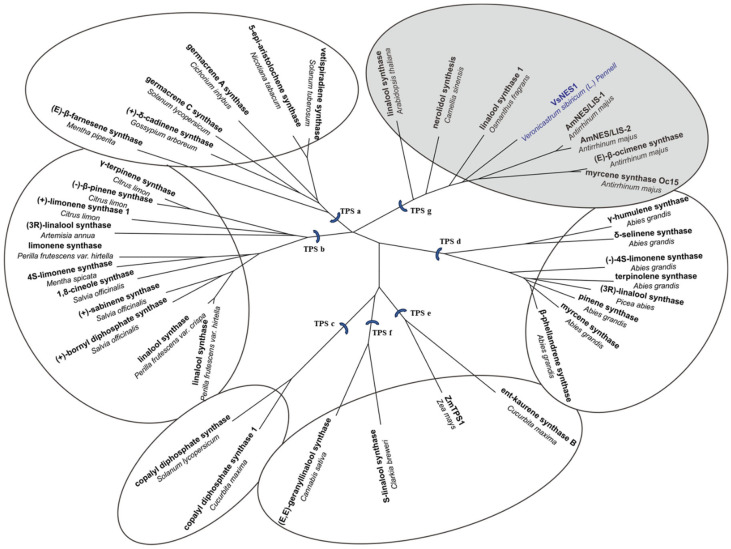
Phylogenetic analysis of VsTPS1. Evolutionary tree of VsTPS1 and related terpene synthases. Species names, protein/common names, and GenBank accession numbers are indicated for each sequence. Details of all sequences included in this analysis are provided in Supplementary Data (File S1). The major plant TPS subfamilies (TPS-a, TPS-b, TPS-c, TPS-d, TPS-e/f, TPS-g) are labeled. Among them, TPS-a primarily contains sesquiterpene synthases; TPS-b typically comprises monoterpene synthases; TPS-c often includes enzymes for diterpene biosynthesis (like copalyl diphosphate synthases); TPS-d is specific to gymnosperms and includes both mono- and diterpene synthases; TPS-e/f and TPS-g contain diterpene synthases and enzymes with diverse functions, respectively. Based on the phylogenetic clustering and subsequent functional characterization, the protein is annotated as *VsNES1* in this study.

The amino acid sequence of VsTPS1 was aligned alongside functionally characterized TPS proteins from *Camellia sinensis*, *Actinidia polygama*, *Osmanthus fragrans*, and *Antirrhinum majus* using MEGA. As illustrated in Supplementary Figure S2, VsTPS1 harbors two characteristic conserved motifs—“DDXXD” and “NSE/DTE”—typical of the TPS family, supporting its classification as a Class I TPS. Notably absent is the “RRX8W” domain, implying that VsTPS1 likely catalyzes the formation of linear rather than cyclized terpenoid products. To elucidate the evolutionary placement of VsTPS1, a phylogenetic tree was generated. VsTPS1 falls within the TPS-g subfamily, most closely related to nerolidol/linalool synthase 1 (AmNES/LIS1) from *Antirrhinum majus*, which belongs to the same plant family (Figure 3). This close relationship suggests that VsTPS1 may also serve a bifunctional role, enabling the synthesis of both linalool and nerolidol in a manner analogous to AmNES/LIS1.

### 2.4. Heterologous Expression and In Vitro Enzymatic Activity of VsTPS1

Gene expression levels were estimated using FPKM (Fragments Per Kilobase per Million mapped reads), a normalization method accounting for gene length and sequencing depth to facilitate reliable cross-tissue comparisons. Analysis of FPKM values indicated that VsTPS1 is expressed in a tissue-specific manner, showing highest abundance in leaves of *V. sibiricum* (Supplementary Figure S5A). The coding sequence of VsTPS1 was cloned into the pET32a vector. After inducing heterologous expression, the target protein was purified via nickel-affinity chromatography, and the fraction with the optimal purification efficiency was selected for subsequent experiments (Supplementary Figure S5B,C).

**Figure 4:**
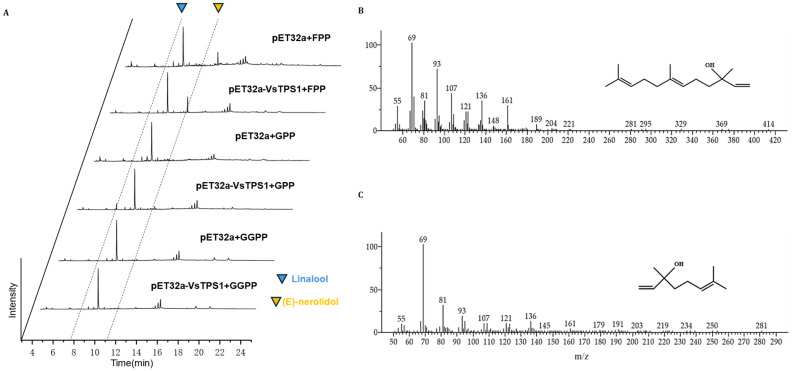
GC-MS analysis of in vitro enzyme activity products of VsTPS1. (**A**) Total ion chromatograms (TICs) of reaction products with FPP, GPP and GGPP substrates. Chromatograms obtained from reactions with the pET32a empty vector crude protein (control) and the purified VsTPS1 recombinant protein are shown. (**B**) Mass spectrum of (E)-nerolidol. (**C**) Mass spectrum of linalool.

The purified VsTPS1 protein was incubated with FPP, GPP, and GGPP as substrates in separate in vitro enzymatic assays. The reaction products were extracted with n-hexane and analyzed by GC–MS. As shown in Figure 4, the control group consisted of crude protein extracted from cells transformed with the empty pET32a vector, while the experimental group contained purified protein from cells expressing pET32a-VsTPS1. When FPP was used as the substrate, a distinct peak emerged at 11.027 min in the pET32a-VsTPS1 group, which was absent in the control. This peak was identified as (E)-nerolidol based on comparison with the NIST mass spectral library, supported by its molecular weight and characteristic ion fragments. With GPP as the substrate, a specific peak at 7.573 min was detected exclusively in the VsTPS1 sample and was identified as linalool. No distinct peaks were observed when GGPP was supplied as the substrate. These results indicate that VsTPS1 is a bifunctional terpene synthase capable of utilizing FPP to produce (E)-nerolidol and GPP to synthesize linalool, resembling the function of AmNES/LIS1 from *Antirrhinum majus*. Based on the respective yields of (E)-nerolidol and linalool, VsTPS1 exhibited significantly higher catalytic efficiency toward FPP than GPP, suggesting that its primary physiological role in *V. sibiricum* may be the biosynthesis of (E)-nerolidol from FPP.

### 2.5. Analysis of VsTPS1 Products in Planta and Heterologous Production in Tobacco

Although VsTPS1 exhibited bifunctional catalytic activity in vitro, we sought to determine its primary enzymatic role in a cellular context. Using a tobacco transient expression system to simulate its physiological environment, we observed that the expression of VsTPS1 in planta (Supplementary Figure S4) led to the production of (E)-nerolidol. In contrast, no linalool was detected—a finding that diverged from the in vitro results and differed significantly from the empty vector control (Supplementary Figure S4).

These results suggest that the primary physiological function of VsTPS1 in *V. sibiricum* is the syn-thesis of (E)-nerolidol. Accordingly, we propose to rename this enzyme as nerolidol synthase 1 (*VsNES1*). The observed functional difference between in vitro and in planta assays may be at-tributed to subcellular localization or other regulatory mechanisms inherent to the plant system.

**Figure 5:**
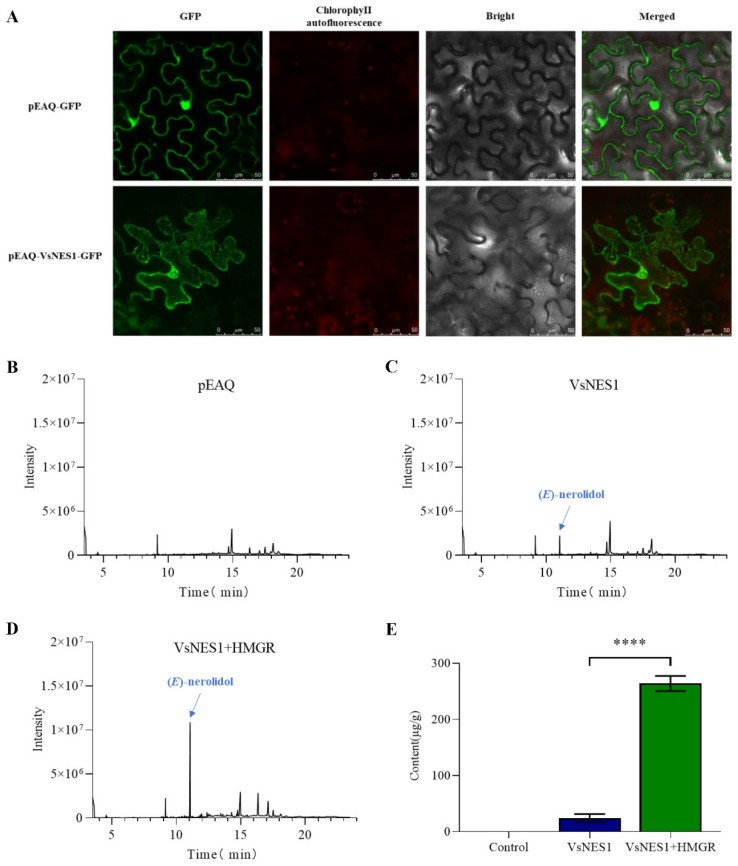
Subcellular localization and trans-nerolidol production analysis. (**A**) Subcellular localization analysis of *VsNES1*. GFP: GFP fluorescence channel; Chlorophyll autofluorescence: Chlorophyll autofluorescence channel; Bright: Bright field; Merged: Merged channel. (**B**) Production of trans-nerolidol in the pEAQ pathway. (**C**) Production of trans-nerolidol in the *VsNES1* pathway. (**D**) Production of trans-nerolidol in the *VsNES1* and *HMGR* co-expression pathway for enhanced terpenoid synthesis. (**E**) Comparison of (E)-nerolidol production levels among the empty vector control, *VsNES1* expression, and *VsNES1*+*HMGR* co-expression groups. (**** *p* < 0.0001).

To determine the subcellular localization of *VsNES1*, a pEAQ-VsNES1-GFP fusion construct was generated and transiently expressed in tobacco leaf epidermal cells, using pEAQ-GFP empty vector as a control. The transfected leaf sections were examined under a confocal microscope (Figure 5A). While GFP fluorescence from the empty vector was observed throughout the cell, including the membrane and nucleus, indicating proper expression, the fluorescence signal from pEAQ-VsNES1-GFP was distinct from chloroplast autofluorescence and predominantly localized to the cytoplasm. This result supports the computational prediction that *VsNES1* is a cytoplasmic protein and consistent with its proposed role in cytosolic terpenoid biosynthesis.

Although heterologous expression of *VsNES1* in tobacco successfully produced (E)-nerolidol, the yield remained relatively low. To enhance the supply of precursor metabolites and increase (E)-nerolidol production—particularly for subsequent functional studies of downstream enzymes—we co-expressed *VsNES1* with *HMGR*, a rate-limiting enzyme in the cytosolic terpenoid pathway, thereby enhancing the metabolic flux through the cytosolic terpenoid biosynthetic pathway. As shown in Figure 5B–E, this metabolic engineering strategy significantly increased the yield of (E)-nerolidol from 24.11 μg/g FW to 263.99 μg/g FW.

## 3. Discussion

The functional characterization of terpene synthases is crucial for understanding the biosynthesis of specialized metabolites in plants [15,21]. In this study, we identified and characterized a terpene synthase, VsTPS1, from *V. sibiricum*. Our comprehensive analyses, including phylogenetic analysis [22,23], heterologous expression [24], and subcellular localization, allow us to elucidate its dual catalytic capability and its primary physiological role in planta.

Initially, in vitro enzyme assays revealed that the purified VsTPS1 protein exhibits bifunctional activity, catalyzing the formation of both (E)-nerolidol from FPP and linalool from GPP (Figure 4). This functional duality is not uncommon in the TPS family, which is relatively conserved, and has been observed in enzymes from other plant species, such as the well-characterized AmNES/LIS1 from *Antirrhinum majus* [25]. The presence of the conserved ‘DDXXD’ and ‘NSE/DTE’ motifs, hallmarks of Class I TPSs [26], and the absence of the ‘RRX8W’ domain associated with cyclization reactions, are consistent with its ability to produce these linear terpenoid products.

However, a critical discrepancy emerged between the in vitro and in planta results. By contrasting the recombinant protein produced both compounds in a test tube, transient expression of VsTPS1 in tobacco leaves resulted in the exclusive production of (E)-nerolidol, with no detectable linalool (Figure 5C). This suggests that the in vitro conditions, which provide unconstrained access to substrates, may not fully replicate the complex cellular environment. We hypothesize that this functional shift is primarily governed by subcellular compartmentalization. Our confocal microscopy analysis confirmed that *VsNES1* is localized exclusively to the cytoplasm (Figure 5A), a finding supported by the lack of a predicted transit peptide. In plants, the substrate FPP for nerolidol synthesis is predominantly available in the cytosol via the mevalonate (MVA) pathway. In contrast, GPP, the precursor for linalool, is primarily synthesized in the plastids via the methylerythritol phosphate (MEP) pathway [9]. The physical separation of the enzyme (cytosolic) from one of its substrates (plastidial GPP) likely explains the absence of linalool detection in the tobacco transient assay. While the recombinant protein demonstrated bifunctional activity in vitro (producing both (E)-nerolidol and linalool), its exclusive production of (E)-nerolidol in planta defines its primary physiological role. Therefore, we have designated this enzyme as VsNES1 (*V. sibiricum* nerolidol synthase 1) to accurately reflect its biological function.

The high expression level of *VsNES1* in the leaves of *V. sibiricum* (Supplementary Figure S5), coupled with its cytosolic localization, strongly indicates that its main physiological role is the synthesis of (E)-nerolidol in this tissue. (E)-Nerolidol is a known volatile sesquiterpene with documented roles in plant defense and indirect resistance by attracting natural enemies of herbivores [26,27,28]. Its production in leaves could be a key defensive strategy for *V. sibiricum*.

The high expression level of in the leaves of *V. sibiricum* (Supplementary Figure S5), coupled with its cytosolic localization, strongly indicates that its primary physiological role is the syn-thesis of (E)-nerolidol in this tissue. (E)-Nerolidol is a well-characterized volatile sesquiterpene that serves as a direct defense metabolite by exhibiting antifungal and antibacterial activities [29,30]. Moreover, it plays a crucial role in indirect defense by attracting natural enemies of herbivores, such as parasitoids and predators, thereby enhancing plant resistance through tritrophic interactions [26,31]. In some plant species, (E)-nerolidol also acts as an herbivore repellent, further contributing to its defensive portfolio (2). Its production in leaves, which are often the primary target of both pathogens and herbivores, could therefore represent a key defensive strategy for *V. sibiricum*.

Finally, our metabolic engineering approach successfully validated the pathway and addressed the limited production of nerolidol in heterologous systems. The drastic increase in (E)-nerolidol yield (from 24.11 to 263.99 μg/g FW) upon co-expression of *VsNES1* with *HMGR* (Figure 5B–E) under-scores that the flux through the cytosolic MVA pathway, and specifically the availability of FPP, is a major bottleneck for sesquiterpene production [32,33]. This strategy of overexpressing a rate-limiting upstream enzyme like *HMGR* to enhance terpenoid accumulation is well-supported by previous studies [34,35]. This result not only confirms the in planta function of *VsNES1* but also provides a feasible strategy for the large-scale production of (E)-nerolidol for further pharmacological or agricultural studies [36].

In conclusion, *VsNES1* is a cytosolic TPS that primarily catalyzes the formation of (E)-nerolidol in planta. Its activity is likely regulated by subcellular substrate availability; this is an elegant example of how metabolic compartmentalization can dictate enzymatic output. The presence of numerous incompletely sequenced TPS genes in our transcriptome highlights that this study merely unveils the first layer of the extensive terpenoid biosynthetic capabilities awaiting discovery in *V. sibiricum*.

## 4. Materials and Methods

### 4.1. Materials

*V. sibiricum* plants were cultivated in the Horticultural West Greenhouse of Shanghai Chenshan Botanical Garden. The culture conditions of plants are similar to those in the wild. In August, roots, stems, leaves and inflorescences that were Pest-free and in good growth condition were collected.

### 4.2. Transcriptome Sequencing Analysis

Total RNA was isolated from multiple tissues of healthy *V. sibiricum* specimens using the MGIEasy RNA Library Prep Kit (BGI, Cat. No. 940-002921-00). The purified RNA was subjected to de novo transcriptome sequencing on the DNBSEQ-T7 platform at BGI. Raw sequencing reads were processed to remove adapter sequences and low-quality bases. Transcript assembly was then performed with Trinity (v2.11.0; https://github.com/trinityrnaseq/trinityrnaseq, accessed on 9 December 2025). To minimize redundancy, transcript clusters were generated using cd-hit (https://github.com/weizhongli/cdhit, accessed on 9 December 2025), and the longest transcript from each cluster was selected as a representative Unigene. Functional annotation of Unigenes was carried out by aligning against public databases such as Nr, Pfam, UniProt, KEGG, GO, KOG, and Pathway. For expression quantification, clean reads from each sample were aligned to the Unigene set using RSEM (https://github.com/deweylab/RSEM, accessed on 9 December 2025), and normalized ex-pression values were calculated as FPKM. Differential gene expression analysis between sample groups was conducted with the DESeq2 package (http://www.bioconductor.org/packages/release/bioc/html/DESeq2.html, accessed on 9 December 2025).

### 4.3. Functional Characterization of VsTPS1

Total RNA was extracted from *V. sibiricum* using the Trizol reagent (Thermo Fisher Scientific, Waltham, MA, USA, Cat. No. 15596018). Subsequently, first-strand cDNA synthesis was performed from the extracted RNA using the HiScript II 1st Strand cDNA Synthesis Kit (Vazyme, Nanjing, China, Cat. No. R201-02). The VsTPS1 gene was amplified using TransStart^®^ FastPfu DNA Polymerase (TransGen, Beijing, China, Cat. No. FS012) and cloned into linearized pET32a via the ClonExpress^®^ Ultra One Step Cloning Kit (Vazyme, Cat. No. C112-02) (Supplementary Table S1). Plasmids were extracted using a TIANGEN Mini Kit (TIANGEN, Beijing, China, Cat. No. DP101-01), and correct insertion was verified by sequencing and NCBI BLAST (version 2.13.0) alignment.

### 4.4. Protein Induction and Purification

The sequenced and verified recombinant plasmid was transformed into *E. coli* Rosetta2 (DE3) competent cells. A single positive clone confirmed by colony PCR was inoculated into 2 mL of LB liquid medium containing 100 μg/mL carbenicillin and cultured overnight at 37 °C. A 2 mL aliquot of the overnight culture was transferred into 100 mL of LB medium with the appropriate antibiotic and incubated at 37 °C with shaking at 200 rpm until the OD_600_ reached 0.6–0.8. The bacterial culture was then placed on ice for 30 min, induced with 0.5 mM IPTG, and further cultured at 16 °C with shaking at 200 rpm for 24 h.

After induction, the cells were harvested by centrifugation at 4 °C and 8000 rpm for 10 min. The pellet was resuspended in 10 mL of Binding Buffer, disrupted by sonication, and centrifuged at 4 °C and 12,000 rpm for 10 min to collect the supernatant. The supernatant was loaded onto a nickel affinity chromatography column pre-equilibrated with Binding Buffer. The column was subsequently washed with 15 mL of Wash Buffer and eluted with 1.5 mL of Elution Buffer, with the eluted fractions being collected. Protein concentration was determined by Bradford assay, and the enzymatic activity was assayed in vitro, with the resulting products analyzed by GC-MS using an Agilent Chem Station equipped with an NIST database.

### 4.5. Heterologous Production of Trans-Nerolidol in Tobacco

The cDNA sequence of the *HMGR* gene of *Salvia miltiorrhiza* was used, together with the VsTPS1 cDNA sequence, to amplify the target genes (Supplementary Table S3). Following the growth of *Escherichia coli* cells harboring the recombinant plasmids pEAQ-VsTPS1 or pEAQ-*HMGR*, positive clones were selected by colony PCR. Plasmids extracted from these clones were verified by sequencing, and the correct constructs were subsequently transformed into Agrobacterium tumefaciens GV3101 (pSoup-p19). Tobacco leaves were transiently transformed according to pre-designed combination of strains (Supplementary Table S4). Four days post-infiltration, compounds were extracted from the transfected leaves for analysis. Specifically, 0.3 g of fresh tobacco tissue was accurately weighed using an analytical balance, extracted with n-hexane for 15 h, and centrifuged. The super-natant was then subjected to GC–MS analysis.

This study employed an Agilent 7890A-5975C GC-MS system equipped with a CYCLODEX-B capillary column (30 m × 0.25 mm × 0.25 μm) for compound detection. High-purity helium was used as the carrier gas at a constant flow rate of 1 mL/min under splitless injection mode with an injection volume of 1 μL. The temperature program was set as follows: initial temperature 50 °C (held for 2 min), ramped at 5 °C/min to 220 °C (held for 10 min). Electron impact (EI) ionization was applied with the ion source temperature set at 230 °C and the quadrupole at 150 °C. The electron energy was 70 eV, and the mass scanning range (m/z) was 50–550. Quantification of (E)-nerolidol was performed by integrating its total ion chromatographic peak area relative to that of the internal standard [37].

### 4.6. Subcellular Localization

The cDNA of *VsNES1* (stop codon excluded) was fused in-frame with the green fluorescent protein (GFP) gene and inserted into the plant expression vector pEAQ via multi-fragment homologous re-combination using specifically designed primers (Supplementary Table S5). A pEAQ construct containing GFP alone served as the control. The verified recombinant plasmid was transformed into *Agrobacterium tumefaciens* GV3101. Positive clones were selected, cultured, and resuspended in 1× infiltration buffer to an OD_600_ of 0.8. The suspensions were infiltrated into the abaxial side of leaves from four-week-old tobacco plants using a needleless syringe. After 48 h, leaf samples were sectioned and examined under a Leica TCS SP8 confocal laser scanning microscope to visualize fluorescence.

## Data Availability

The original contributions presented in this study are included in the article. The raw sequencing data have been deposited in the Genome Sequence Archive (GSA) at the China National Center for Bioinformation (CNCB) under accession number CRA034476 (https://ngdc.cncb.ac.cn/gsa, accessed on 9 December 2025). Further inquiries can be directed to the corresponding author.

## Supplementary Materials

The following supporting information can be downloaded at: https://www.mdpi.com/article/10.3390/plants14243813/s1. Table S1: Primers used for cloning of VsTPS1; Table S2: Enzyme-Catalyzed Reaction System; Table S3: Table of homologous recombination primers; Table S4: Tobacco Leaf Transient Transformation System; Table S5: Subcellular localization primer table; Table S6: Statistics of sequencing data volume; Table S7: Gene statistics of terpenoid metabolic pathways; Table S8: Basic physicochemical properties of VsTPS1; Figure S1: Analysis of terpenoid synthesis-related genes; Figure S2: Multiple sequence alignment results, Figure S3: Prediction of transmembrane domain, signal peptide, and secondary structure of VsTPS1, Figure S4. GC-MS chromatogram peaks of VsTPS1 tobacco heterologous; Figure S5. Expression and purification analysis of VsTPS1; File S1: The data source for the tree diagram in Figure 3.

## References

[1] Pang S.Y. (2018). Literature records and functional evolution of Clematidis Radix et Rhizoma. Guid. J. Tradit. Chin. Med. Pharmacy..

[2] Effah E., Svendsen L., Barrett D.P., Clavijo McCormick A. (2022). Exploring plant volatile-mediated interactions between native and introduced plants and insects. Sci. Rep..

[3] Satta A., Lu Z., Plan M.R., Esquirol L., Ebert B.E. (2022). Microbial Production, Extraction, and Quantitative Analysis of Isoprenoids. Methods Mol. Biol..

[4] Shi Y.C., Yu Y.X., Gao J.X., Wang X., Shang X.Y., Xu J. (2025). Iridoid glycoside dimers from fruits of Cornus officinalis and their anti-inflammatory activity. Front. Chem..

[5] Li W., Wang X., Chen Y., Ding Y., Ling X., Yuan B., Tao J. (2024). Luteolin-7-O-glucoside promotes macrophage release of IFN-β by maintaining mitochondrial function and corrects the disorder of glucose metabolism during RSV infection. Eur. J. Pharmacol..

[6] Teng J., Li H.Q., Yao Z., Zhang Y.W., Zhang F.G., Duan H.Q. (2008). Study on antitumor diterpenoids from Veronica sibirica. Chin. Tradit. Herb. Drugs..

[7] Zeng X., Guo F., Ouyang D. (2020). A review of the pharmacology and toxicology of aucubin. Fitoterapia.

[8] Tsuruta H., Paddon C.J., Eng D., Lenihan J.R., Horning T., Anthony L.C., Regentin R., Keasling J.D., Renninger N.S., Newman J.D. (2009). High-level production of amorpha-4,11-diene, a precursor of the antimalarial agent artemisinin, in *Escherichia coli*. PLoS ONE.

[9] Vranová E., Coman D., Gruissem W. (2013). Network analysis of the MVA and MEP pathways for isoprenoid synthesis. Annu. Rev. Plant Biol..

[10] Baeshen M.N., Al-Hejin A.M., Bora R.S., Ahmed M.M., Ramadan H.A., Saini K.S., Baeshen N.A., Redwan E.M. (2015). Production of Biopharmaceuticals in *E. coli*: Current Scenario and Future Perspectives. J. Microbiol. Biotechnol..

[11] Huang S., Zhang Y., Wei X., Cai H., Wu Z., Su Z., Ma Z. (2025). Chromosome-level genome assembly of an important ethnic medicinal plant *Callicarpa nudiflora*. Sci. Data..

[12] Zhu X., Liu X., Liu T., Wang Y., Ahmed N., Li Z., Jiang H. (2021). Synthetic biology of plant natural products: From pathway elucidation to engineered biosynthesis in plant cells. Plant Commun..

[13] Lichman B.R. (2021). The scaffold-forming steps of plant alkaloid biosynthesis. Nat. Prod. Rep..

[14] Jiang F., Gong T., Chen J., Chen T., Yang J., Zhu P. (2021). Synthetic biology of plants-derived medicinal natural products. Sheng Wu Gong Cheng Xue Bao.

[15] Tholl D. (2015). Biosynthesis and biological functions of terpenoids in plants. Adv. Biochem. Eng. Biotechnol..

[16] Hashimoto H., Uragami C., Cogdell R.J. (2016). Carotenoids and Photosynthesis. Subcell. Biochem..

[17] Hillier S.G., Lathe R. (2019). Terpenes, hormones and life: Isoprene rule revisited. J. Endocrinol..

[18] Cheng T., Wang L., Sun C., Xie C. (2022). Optimizing the downstream MVA pathway using a combination optimization strategy to increase lycopene yield in *Escherichia coli*. Microb. Cell Fact..

[19] Tan J., Zhang C., Pai H., Lu W. (2022). Heterologous biosynthesis of taraxerol by engineered Saccharomyces cerevisiae. FEMS Microbiol. Lett..

[20] Cao L., Li J., Yang Z., Hu X., Wang P. (2023). A review of synthetic biology tools in *Yarrowia lipolytica*. World J. Microbiol. Biotechnol..

[21] Chen F., Tholl D., Bohlmann J., Pichersky E. (2011). The family of terpene synthases in plants: A mid-size family of genes for specialized metabolism that is highly diversified throughout the kingdom. Plant J..

[22] Jia Q., Li G., Köllner T.G., Fu J., Chen X., Xiong W., Crandall-Stotler B.J., Bowman J.L., Weston D.J., Zhang Y. (2016). Microbial-type terpene synthase genes occur widely in nonseed land plants, but not in seed plants. Proc. Natl. Acad. Sci. USA.

[23] Karunanithi P.S., Zerbe P. (2019). Terpene Synthases as Metabolic Gatekeepers in the Evolution of Plant Terpenoid Chemical Diversity. Front. Plant Sci..

[24] Dickschat J.S. (2016). Bacterial terpene cyclases. Nat. Prod. Rep..

[25] Nagegowda D.A., Gutensohn M., Wilkerson C.G., Dudareva N. (2008). Two nearly identical terpene synthases catalyze the formation of nerolidol and linalool in snapdragon flowers. Plant J..

[26] Tholl D. (2006). Terpene synthases and the regulation, diversity and biological roles of terpene metabolism. Curr. Opin. Plant Biol..

[27] Kappers I.F., Aharoni A., van Herpen T.W., Luckerhoff L.L., Dicke M., Bouwmeester H.J. (2005). Genetic engineering of terpenoid metabolism attracts bodyguards to Arabidopsis. Science.

[28] Brosset A., Blande J.D. (2022). Volatile-mediated plant-plant interactions: Volatile organic compounds as modulators of receiver plant defence, growth, and reproduction. J. Exp. Bot..

[29] Chen S., Zhang L., Cai X., Li X., Bian L., Luo Z., Li Z., Chen Z. (2020). (E)-Nerolidol is a volatile signal that induces defenses against insects and pathogens in tea plants. Hortic. Res..

[30] De Moura D.F., Rocha T.A., De Melo Barros D., Da Silva M.M., Dos Santos Santana M., Neta B.M., Cavalcanti I.M.F., Martins R.D., Da Silva M.V. (2021). Evaluation of the antioxidant, antibacterial, and antibiofilm activity of the sesquiterpene nerolidol. Arch. Microbiol..

[31] Mutinda E.S., Mkala E.M., Ren J., Kimutai F., Waswa E.N., Odago W.O., Nanjala C., Gichua M.K., Njire M.M., Hu G.W. (2023). A review on the traditional uses, phytochemistry, and pharmacology of the genus Veronicastrum (Plantaginaceae). J. Ethnopharmacol..

[32] Wu S., Schalk M., Clark A., Miles R.B., Coates R., Chappell J. (2006). Redirection of cytosolic or plastidic isoprenoid precursors elevates terpene production in plants. Nat Biotechnol..

[33] George K.W., Alonso-Gutierrez J., Keasling J.D., Lee T.S. (2015). Isoprenoid drugs, biofuels, and chemicals--artemisinin, farnesene, and beyond. Adv. Biochem. Eng. Biotechnol..

[34] Wang C., Liwei M., Park J.-B., Jeong S.-H., Wei G., Wang Y., Kim S.-W. (2018). Microbial Platform for Terpenoid Production: *Escherichia coli* and Yeast. Front. Microbiol..

[35] Diamond A., Desgagné-Penix I. (2016). Metabolic engineering for the production of plant isoquinoline alkaloids. Plant Biotechnol. J..

[36] Chan W.K., Tan L.T., Chan K.G., Lee L.H., Goh B.H. (2016). Nerolidol: A Sesquiterpene Alcohol with Multi-Faceted Pharmacological and Biological Activities. Molecules.

[37] Tamogami S., Takahashi Y., Abe M., Noge K., Rakwal R., Agrawal G.K. (2011). Conversion of airborne nerolidol to DMNT emission requires additional signals in *Achyranthes bidentata*. FEBS Lett..

