# Supplementary material for: Characterization of Nerolidol Synthase (VsNES1) from Veronicastrum sibiricum via Transcriptome Analysis
Source: Plants (Basel). 2025 Dec 15;14(24):3813. doi: 10.3390/plants14243813 (PMC12736513; doi:10.3390/plants14243813)
Supplement: Supplementary file 1 [file plants-14-03813-s001.zip › plants-3996221-supplementary.pdf]

Supplementary Table S1. Primers used for cloning of *VsTPS1*

Primer	5' to 3'
VsTPS1-F	CCATGGCTGATATCGGATCCGAATATGGCAGTTCGAAACAGTAG
VsTPS1-R	GTGGTGGTGGTGCTCGAGTGCCTAATCCAAGGATGTCTGAT

Supplementary Table S2. Enzyme-Catalyzed Reaction System

Group	Enzymatic reaction system (500 μL) was prepared with three biological replicates per combination.
1	20 μg purified recombinant protein + 40 μM FPP + 25 mM HEPES (pH 7.0) + 5 mM $MgCl_2$ + 5 mM DTT
2	pET32a empty vector crude protein + 40 μM FPP + 25 mM HEPES (pH 7.0) + 5 mM $MgCl_2$ + 5 mM DTT
3	20 μg purified recombinant protein + 40 μM GPP + 25 mM HEPES (pH 7.0) + 5 mM $MgCl_2$ + 5 mM DTT
4	pET32a empty vector crude protein + 40 μM GPP + 25 mM HEPES (pH 7.0) + 5 mM $MgCl_2$ + 5 mM DTT
5	20 μg purified recombinant protein + 40 μM GGPP + 25 mM HEPES (pH 7.0) + 5 mM $MgCl_2$ + 5 mM DTT
6	pET32a empty vector crude protein + 40 μM GGPP + 25 mM HEPES (pH 7.0) + 5 mM $MgCl_2$ + 5 mM DTT

Supplementary Table S3. Table of homologous recombination primers

Primer	5' to 3'
VsTPS1-F	TTCTGCCCAAATTCGCGACCGGTATGGCAGTTCGAAACAGTAG
VsTPS1-R	GAGTTAAAGGCCTCGAGTCACTAATCCAAGGATGTCTGAT
HMGR-F	TTCTGCCCAAATTCGCGACCGGTATGGATATCCGCCGGAGGCCAGCCC
HMGR-R	GAGTTAAAGGCCTCGAGTCAGGAGCCAATCTTCGTGATGTCCCTG

Supplementary Table S4. Tobacco Leaf Transient Transformation System

No	Group
1	pEAQ
2	pEAQ-VsTPS1
3	pEAQ-VsTPS1 + pEAQ-HMGR

Supplementary Table S5. Subcellular localization primer table

Primer	5' to 3'
VsNES1gfp-F	TTCTGCCCAAATTCGCGACCGGTATGGCAGTTCGAAACAGTAG

VsNES1gfp-R	TCTACCATGCTTCCGCTTCCATCCAAGGATGTCTGATTGG
GFPgsgs-F	GGAAGCGGAAGCATGGTAGATCTGACTAGTAAAGGAG
GFP-R	GAGTTAAAGGCCTCGAGTCAGCTAGCTTTGTATAGTTCATCCATG

Supplementary Table S6. Statistics of sequencing data volume

Sample	Raw reads	Raw bases	Clean reads	Clean bases	Q20 rate	Q30 rate	GC content
Vsi-F1	43,370,340	6,505,551,000	43,341,924	5,952,446,756	98.928%	96.843%	45.284%
Vsi-F2	43,334,022	6,500,103,300	43,304,936	5,916,854,238	99.021%	97.113%	45.404%
Vsi-F3	43,349,490	6,502,423,500	43,320,018	5,932,589,156	98.993%	97.049%	45.477%
Vsi-L1	43,361,826	6,504,273,900	43,332,694	6,035,662,686	99.023%	97.086%	46.216%
Vsi-L2	43,344,826	6,501,723,900	43,315,164	6,018,784,446	98.957%	96.934%	46.117%
Vsi-L3	43,365,798	6,504,869,700	43,335,676	6,046,353,142	98.977%	97.008%	46.141%
Vsi-R1	43,398,180	6,509,727,000	43,367,556	6,057,921,148	99.045%	97.189%	45.497%
Vsi-R2	43,372,440	6,505,866,000	43,317,886	6,101,417,176	98.965%	96.974%	45.701%
Vsi-R3	43,366,710	6,505,006,500	43,313,606	6,116,051,598	98.903%	96.783%	45.582%
Vsi-S1	43,388,596	6,508,289,400	43,342,672	6,091,822,846	98.894%	96.792%	45.863%
Vsi-S2	43,365,244	6,504,786,600	43,336,278	6,012,398,456	98.888%	96.740%	45.630%
Vsi-S3	43,367,204	6,505,080,600	43,313,716	6,045,205,812	98.981%	97.005%	45.517%

Note: Sample is the sample name; Raw reads is the number of reads of raw unfiltered data; Raw bases are the base numbers of unfiltered raw data; Clean reads is the number of reads remaining after filtering; The number of alkali residues remaining after filtering the clean bases; Q20 rate refers to the proportion of bases with a quality value greater than 20 (error rate less than 1%) in the total sequence after filtering. The higher the Q20 rate, the higher the sequencing quality; Q30 rate refers to the proportion of bases with a mass value greater than 30 (error rate less than 0.1%) in the total sequence after filtering. The higher the Q30 rate, the higher the sequencing quality; GC content is the percentage of GC content in the filtered data.

Supplementary Table S7. Gene statistics of terpenoid metabolic pathways

Pathway	Gene	KO	number
MEP pathway	1-Deoxy-D-xylulose 5-phosphate synthase (DXS)	K01662	6
	1-Deoxy-d-xylulose 5-phosphate reductoisomerase (DXR)	K00099	3
	2-C-methyl-d-erythritol 4-phosphatecytidylyltransferase (MCT)	K01770	1
	4-(Cytidine 5-diphospho)-2-C-methyl-d-erythritol kinase (CMK)	K00991	3
	2-C-methyl-d-erythritol 2,4-cyclodiphosphate synthase (MDS)	K00919	1
	4-Hydroxy-3-methylbut-2-enyl-diphosphate synthase (HDS)	K03526	3

	4-Hydroxy-3-methylbut-2-en1-yl diphosphate reductase (HDR)	K03527	4
MVA pathway	Acetyl-CoA acetyl transferase (AACT)	K14175	11
	3-Hydroxy-3-methylglutaryl-CoA synthase (HMGS)	K01641	12
	3-Hydroxy-3-methylglutaryl-CoA reductase (HMGR)	K00021	5
	Mevalonate kinase (MK)	K00869	2
	Phosphomevalonate kinase (PMK)	K00938	3
	Diphosphomevalonate decarboxylase (MVD)	K01597	4
	Isopentenyl-diphosphate delta-isomerase (IDI)	K01823	3
Important Branches	Farnesyl diphosphate synthase (FPPS)	K00787	3
	Geranyl diphosphate synthase (GPPS)	K14066	5
	Geranylgeranyl diphosphate synthetase (GGPPS)	K13789	14
	Monoterpenoid biosynthesis	ko00902	31
	Sesquiterpenoid and triterpenoid biosynthesis	ko00909	32
	Diterpenoid biosynthesis	ko00904	24
	Carotenoid biosynthesis	ko00906	58

Supplementary Table S8. Basic physicochemical properties of VsTPS1 protein

Gene ID	Name	Sequence Length /aa	Protein Molecular Weight /kDa	Theoretical PI value	Instability coefficient	Average coefficient of hydrophilicity	Secondary Structure			
							Alpha helix	Beta turn	Random coil	Extended strand
VsibChr2G071430	VsTPS1	532	61.04	5.44	33.22	-0.364	68.61%	0%	30.08%	0.56%

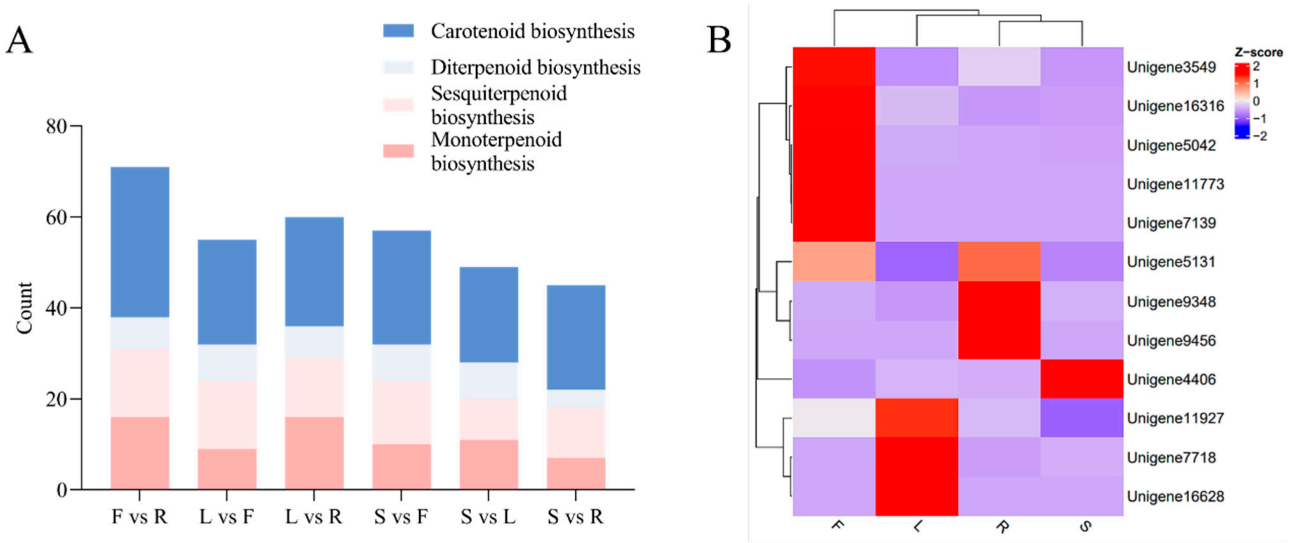

Supplementary Figure S1. Analysis of terpenoid synthesis-related genes (F, flower; L, leaf; R, root; S, stem). A, Statistical analysis of the differences in the number of genes related to terpenoid synthesis across various terpenoid biosynthesis pathways. The x-axis represents the sample groups, and the y-axis represents the gene count. B, Heatmap showing the expression levels of candidate VsTPS genes. The color scale represents expression levels, with red indicating higher expression and blue indicating lower expression.

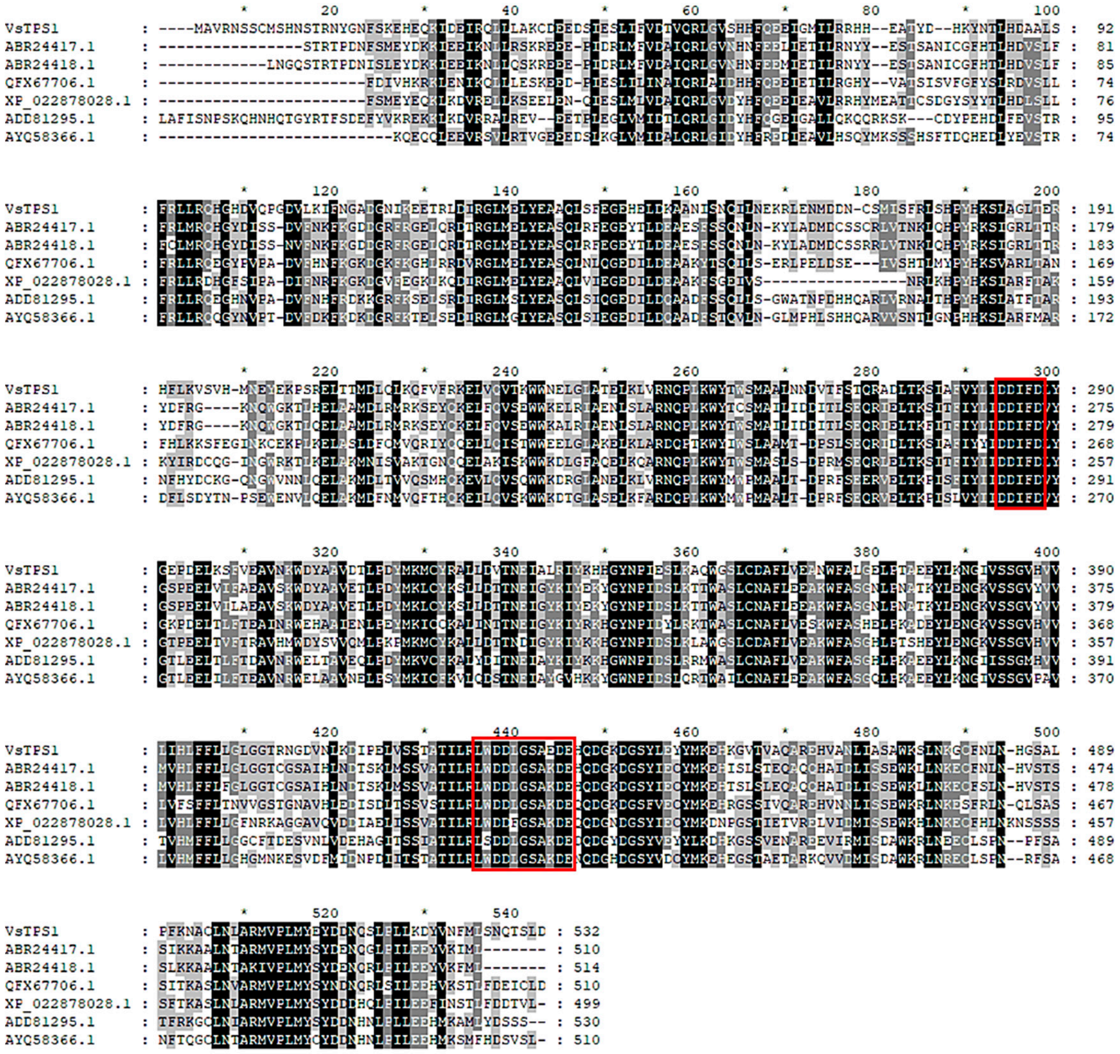

Supplementary Figure S2. Multiple sequence alignment results. Identification of conserved domains in TPS. The red boxes highlight the conserved domains "DDXXD" and "NSE/DTE". Accession numbers and species origins of the aligned sequences are as follows: ABR24417.1 (*Antirrhinum majus* nerolidol/linalool synthase 1), ABR24418.1 (*Antirrhinum majus* nerolidol/linalool synthase 2), QFX67706.1 (*Osmanthus fragrans* linalool synthase 1), XP_022878028.1 (*Olea europaea* var. *sylvestris* (3S,6E)-nerolidol synthase 1-like), ADD81295.1 (*Actinidia polygama* linalool synthase), and AYQ58366.1 (*Camellia sinensis* nerolidol synthase).

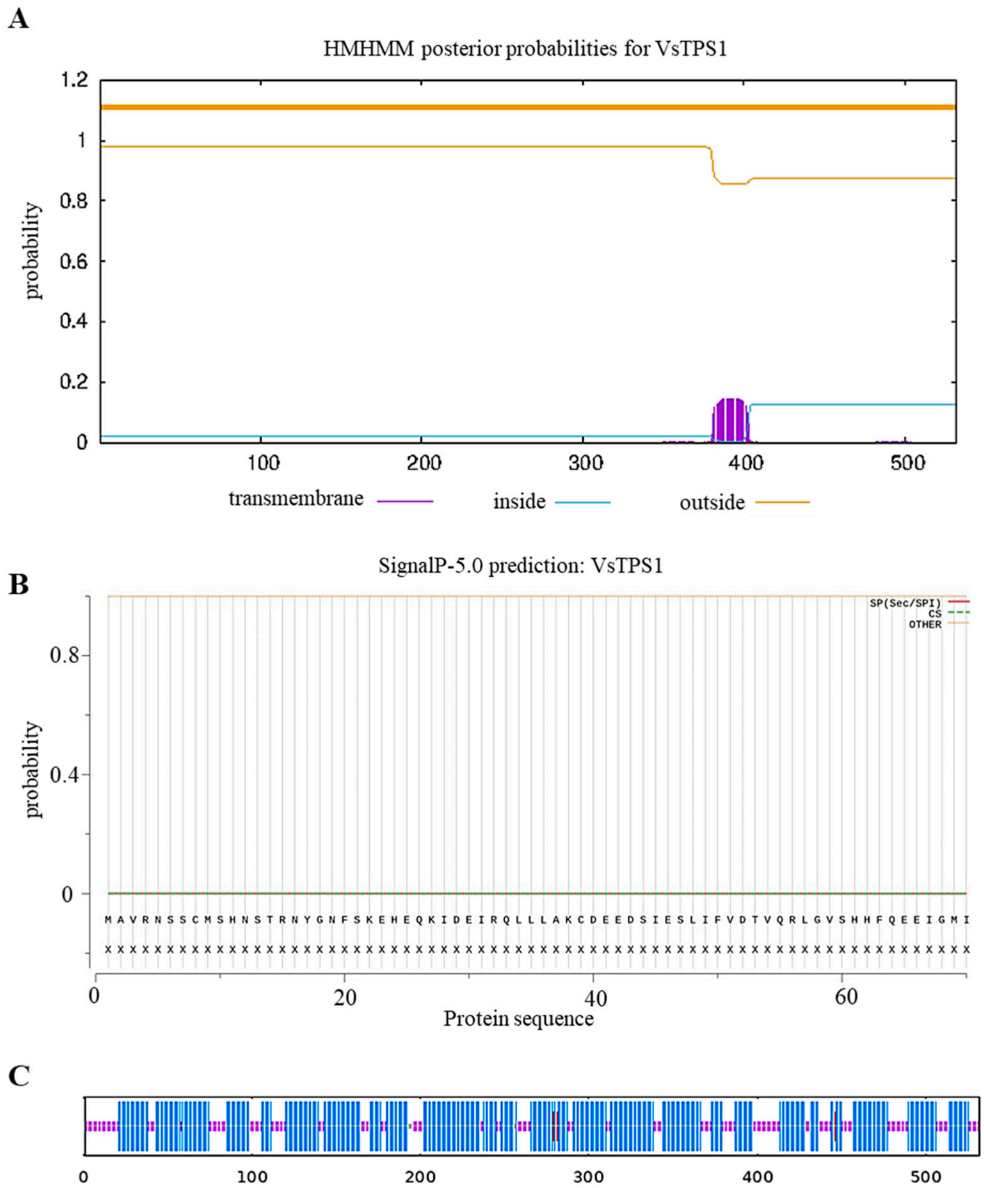

Supplementary Figure S3. Prediction of transmembrane domain, signal peptide, and secondary structure of VsTPS1. A, Transmembrane domain prediction of VsTPS1. B, Signal peptide prediction of VsTPS1. C, Secondary structure prediction of VsTPS1.

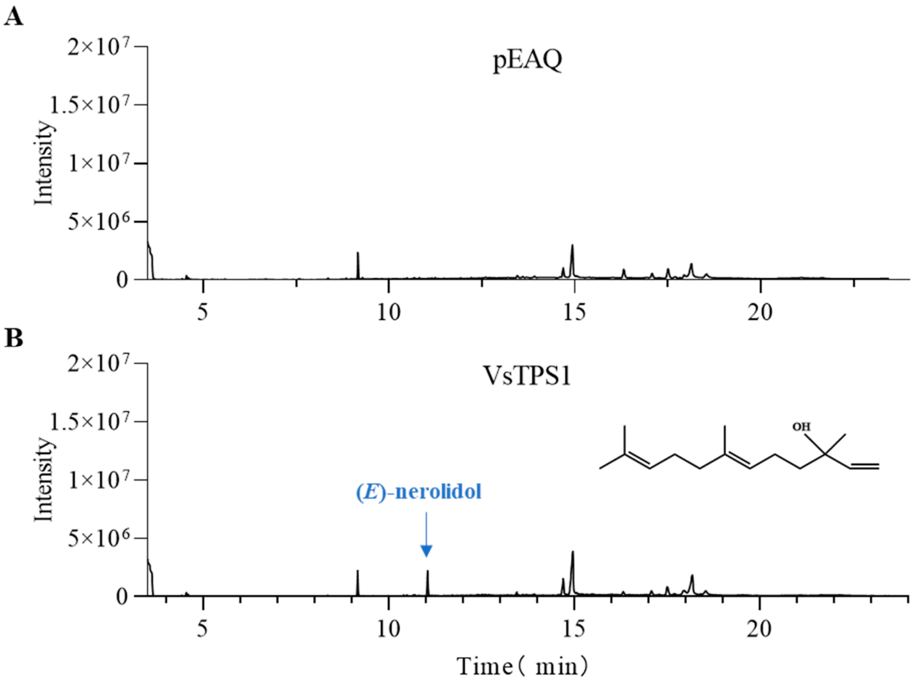

Supplementary Figure S4. GC-MS chromatogram peaks of VsTPS1 tobacco heterologous. A, Control chromatogram from tobacco leaves transformed with the empty vector. No peak was detected at the retention time of 11.1 min. B, Chromatogram from tobacco leaves expressing VsTPS1. A distinct peak was observed at a retention time of 11.1 min, which was identified as (E)-nerolidol by comparison with the NIST mass spectral library and authentic standards.

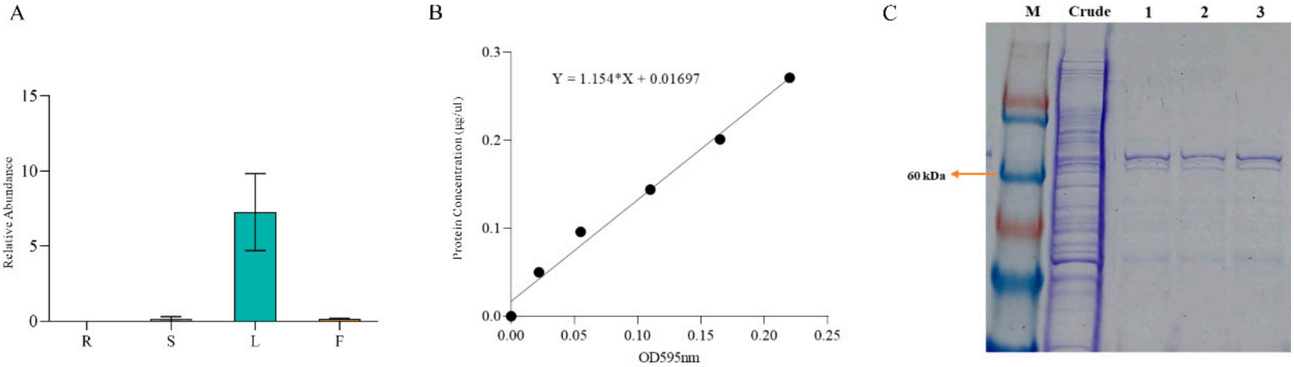

Supplementary Figure S5. Expression and purification analysis of VsTPS1. A, Expression levels of *VsTPS1* in different tissues. B, BSA standard curve. C, SDS-PAGE analysis of VsTPS1 protein purification. M: Protein marker; Crude: Crude supernatant of VsTPS1; Lanes 1, 2, 3: Purified fractions of VsTPS1 crude supernatant.
